# Is the Second Sodium Pump Electrogenic?

**DOI:** 10.1155/2013/698674

**Published:** 2012-12-26

**Authors:** L. E. Thomas, M. A. Rocafull, J. R. del Castillo

**Affiliations:** Laboratorio de Fisiología Molecular, Centro de Biofísica y Bioquímica, Instituto Venezolano de Investigaciones Científicas (IVIC), Apartado 20632, Caracas 1020 Districo Capital, Venezuela

## Abstract

Transepithelial sodium transport is a process that involves active Na^+^ transport at the basolateral membrane of the epithelial cell. This process is mediated by the Na^+^/K^+^ pump, which exchanges 3 internal Na^+^ by 2 external K^+^ inducing a net charge movement and the second Na^+^ pump, which transports Na^+^ accompanied by Cl^−^ and water. It has been suggested that this pump could also be electrogenic. Herein, we evaluated, in MDCK cells, the short-circuit current (*I*_sc_) generated by these Na^+^ pumps at the basolateral membrane of the epithelial cells, using amphotericin B as an apical permeabilizing agent. In Cl^−^-containing media, *I*_sc_ induced by amphotericin B is totally inhibited by ouabain, indicating that only the electrogenic Na^+^/K^+^ pump is detectable in the presence of Cl^−^. Electrogenicity of the second Na^+^ pump can be demonstrated in Cl^−^-free media. The existence of a furosemide-sensitive component of *I*_sc_, in addition to an ouabain-sensitive one, was identified in absence of chloride. Passive Cl^−^ movement associated with the function of the second Na^+^ pump seems to be regulated by the pump itself. These results demonstrate that the second Na^+^ pump is an electroneutral mechanism result from the stoichiometric movement of Na^+^ and Cl^−^ across the basolateral plasma membrane of the epithelial cell.

## 1. Introduction

In epithelia, the transcellular movement of sodium is a two-step process, which is dependent on cellular energy and involves the participation of transporters. The sodium ion enters the epithelial cell across the apical pole of the cell following its electrochemical gradient and is actively extruded across the basolateral plasma membrane. There is an apical electrogenic movement of sodium ions with no direct coupling to the movement of other solutes through sodium channels [1], in addition to the sodium movement coupled with water-soluble organic solutes, such as hexoses, and amino acids [2–4]. Moreover, there is a coupled movement of Na^+^ and Cl^−^ across the brush-border membrane that results from two processes located at the apical membranes of the epithelial cells, exchange of Na^+^ and H^+^, and exchange of Cl^−^ and HCO_3_^−^. The bicarbonate and hydrogen ions are formed intracellularly from H_2_CO_3_ generated by the action of carbonic anhydrase. The downhill movement of sodium leads to a loss of H^+^, through Na^+^/H^+^ exchange. The two exchange systems are interrelated and controlled by the intracellular pH [5]. Finally, the sodium liberated into the cytoplasm is then actively pumped out of the cell across the basolateral plasma membrane, thus maintaining the electrochemical gradient for this ion across the plasma membrane of the epithelial cells. The active epithelial absorption of sodium is responsible for the maintenance of a small but significant transepithelial potential difference of 3–5 mV (serosa positive), which drives diffusional flux of chloride and water from the mucosal to the serosal fluid, either across the tight junctions or possibly also across the cell. In the small intestine and the proximal tubule, the primary active Na^+^ transport across the basolateral plasma membrane of the epithelial cells has been mainly attributed to the Na^+^/K^+^ pump. However, in these epithelia, active Na^+^ transport seems to be not exclusively mediated by the Na^+^/K^+^ exchange pump. In the proximal tubule, two different mechanisms for sodium transport across the basolateral plasma membrane of the cell have been described and characterized [6–8]. One mechanism exchanges the intracellular sodium for the extracellular potassium, whilst the other actively expels sodium, the cation being followed passively by chloride and water. The first of these mechanisms is inhibited by ouabain, weakly inhibited by ethacrynic acid, and insensitive to furosemide and triflocin, whereas the second mechanism is refractory to ouabain but inhibited by ethacrynic acid, furosemide, and triflocin. Both mechanisms are dependent on cellular energy since both are suppressed by 2,4-dinitrophenol or anoxia and derive their energy from the hydrolysis of ATP. In the rabbit ileum, Nellans and Schultz [9] were unable to detect a direct relationship between the ouabain-sensitive Na^+^/K^+^ exchange mechanism and the active transepithelial Na^+^ transport. Additionally, they reported that the maintenance of cell volume did not appear to be dependent upon the ouabain-sensitive Na^+^/K^+^ pump. These results suggest the existence of K^+^-independent active Na^+^ extrusion in the small intestine. In this sense, two different mechanisms for active Na^+^ transport across basolateral plasma membrane have been described in this tissue [10]. These Na^+^ transport mechanisms have been associated with two different ATPase activities, also present in both renal and intestinal tissues [11–15]. The Na^+^/K^+^-ATPase activity that requires K^+^ to function is inhibited by ouabain and vanadate but is insensitive to furosemide. In contrast, The Na^+^-ATPase does not require K^+^, is insensitive to ouabain, but inhibited by furosemide and vanadate. This K^+^-independent, ouabain-insensitive mechanism has been denominated as the second sodium pump and has been implicated in isosmotic cell volume regulation [7, 8]. Recently, the intestinal K^+^-independent, ouabain-insensitive Na^+^-ATPase has been isolated, purified, and cloned [16], and its biochemical and physiological characteristics have been reviewed [17].

It is widely accepted that the Na^+^/K^+^ pump is electrogenic. However, there are no conclusive evidences about the electrogenicity of the K^+^-independent, ouabain-insensitive Na^+^ pump. In this sense, Proverbio and Whittembury [18], working with kidney cortex slices and measuring the cell's electrical potential during active Na^+^ extrusion, in the presence of ouabain, were able to demonstrate the transfer of electric charges across the plasma membrane of the tubular cells mediated by an ethacrynic acid-sensitive mechanism, suggesting that the Na^+^ transport by the second sodium pump could be electrogenic. In this paper, we evaluated this possibility using polarized epithelial cells grown on permeable supports and measuring short-circuit currents (*I*_sc_) in the Ussing chambers. The use of amphotericin B to permeabilize the apical membranes of epithelial cells to small cations allowed us to measure the currents generated at the basolateral plasma membrane by the Na^+^ pumps.

## 2. Materials and Methods 

### 2.1. Materials

Media were prepared from deionized water (18 MΩ-cm) and analytical grade reagents. Ouabain, furosemide, amphotericin B, Dulbecco's modified Eagle's medium (DMEM), penicillin/streptomycin, HEPES, and trypsin were from Sigma Chemical. Fetal calf serum (FCS) was from Life Technologies. 

### 2.2. Methods

#### 2.2.1. Cell Culture

Madin-Derby canine kidney (MDCK) cells (type II) from American Type Culture Collection were suspended in DMEM supplemented with 10% FCS, glutamine (2 mM), penicillin (50 IU/mL), and streptomycin (50 mg/mL) and were plated on Transwell permeable supports (with pore of 0.4 *μ*m and diameter of 12 mm; Corning Incorporated) at a seeding density of 10^6^ cells/cm^2^. Cells were maintained at 37°C in air-5% CO_2_ until they reached confluence (typically on day 6–8 after plating). Formation of tight monolayers was tested by measuring transepithelial resistance and transepithelial potential difference. 

#### 2.2.2. Isolation of Cell Cultured Membrane Fraction

To determine the Na^+^-ATPase activity in MDCK cells, they were homogenized in cold solution A (250 mM sucrose, 20 mM Tris-HCl pH 7.2, 1 mM EDTA, and 1 mM PMSF) and then centrifuged at 5000 ×g for 10 min at 4°C. Supernatant was ultracentrifuged at 100,000 ×g for 1 h at 4°C. The pellet, containing the microsomal fraction, was resuspended in 500 *μ*L of solution B (20 mM Tris-HCl pH 7.25 and 1 mM PMSF) and the ATPase activities were immediately determined. 

#### 2.2.3. ATPase Activities' Determination

The ATPase activities were determined as described [11, 19] and expressed in nmol of phosphate liberated per mg of protein per minute. ATPase activity determined in the presence of Mg^2+^ alone is referred as Mg^2+^-ATPase. The difference in activity between the Mg^2+^-ATPase and in the presence of both magnesium and sodium is denoted as the Na^+^-ATPase. Both Mg^2+^- and Na^+^-ATPases are insensitive to 1 mM ouabain. The difference in activity, obtained in the presence of Mg^2+^, Na^+^, K^+^, and ouabain and the activity determined in the presence of Mg^2+^ Na^+^, and K^+^ is considered to be due to the Na^+^/K^+^-ATPase. 

#### 2.2.4. Protein Determination

The membrane protein was determined as described [20], with bovine serum albumin as standard. 

#### 2.2.5. RNA Isolation and cDNA Synthesis

Total RNA was prepared from MDCK cell monolayer at 100% confluence. Single-strand cDNA was synthesized from 3 *μ*g of total RNA, using oligo(dT)_20_ and the ThermoScript RT-PCR System (Invitrogen). 

#### 2.2.6. Identification of the ATNA Gene (Na^+^-ATPase) in MDCK Cells

The mRNA expression of the Na^+^-ATPase *α*-subunit (atna) was determined by specific RT-PCRs as described [21], employing the primers NAS (5′-CTGCCTATCCT-TAAGCTGTCCA-3′) and CAS (5′-TCAAAGGACTTCCCAAGGTCAAACTGTG), at a final concentration of 400 and 200 nM, respectively. Polymerase chain reaction was carried out employing Platinum Taq DNA polymerase (Invitrogen). Cycling parameters were 94°C for 2 min; followed by 32 cycles at 94°C for 1 min, 55°C for 1 min, and 68°C for 1 min; then followed by 68°C for 10 min. The RT-PCR products were analyzed by 2% agarose gel electrophoresis and stained with 0.5 *μ*g/mL of ethidium bromide. 

#### 2.2.7. Electrical Measurements in the Ussing Chamber

Cell monolayers were typically used on days 6–8 after plating. Once mounted in the chamber, cell monolayers were bathed with media composed of (in mM) 110 NaCl, 5 KCl, 7.5 K_2_SO_4_, 1 MgSO_4_, 1.6 CaCl_2_, 1 K_2_HPO_4_, 10 glucose, 5 glutamine, and 20 HEPES, pH 7.4, at 25°C. In the Cl^−^-free media, chloride was substituted by gluconate. To measure transport across the basolateral membranes, amphotericin B was added to the apical compartment at a final concentration of 10 *μ*M. An automated voltage clamp unit (voltage-current clamp VCC 600, Physiological Instruments) was used to continuously monitor the transepithelial potential difference (Pd) and the short-circuit current (*I*_sc_). All measurements were made at 25°C. Cell monolayers were kept under open-circuit conditions for about 15 min during equilibration to the medium. The epithelium was then short circuited by clamping the transepithelial potential to 0 mV, and *I*_sc_ was continuously displayed, digitized, and stored for analyses. A voltage pulse of 1 mV during 1 sec every 90 sec was applied to the preparation to estimate the transepithelial resistance. Ouabain (1 mM) was added to the basolateral side for inhibition of the Na^+^/K^+^-pump. Furosemide (3 mM) was also added to the basolateral side for inhibition of the Na^+^ pump. Rotenone (10 *μ*M) was added to the basolateral side for metabolic inhibition. 

#### 2.2.8. Statistics

Results are presented as the means ± SEM. The difference between means was evaluated by analysis of variance and considered significant at *P* < 0.05.

## 3. Results

**Figure 1:**
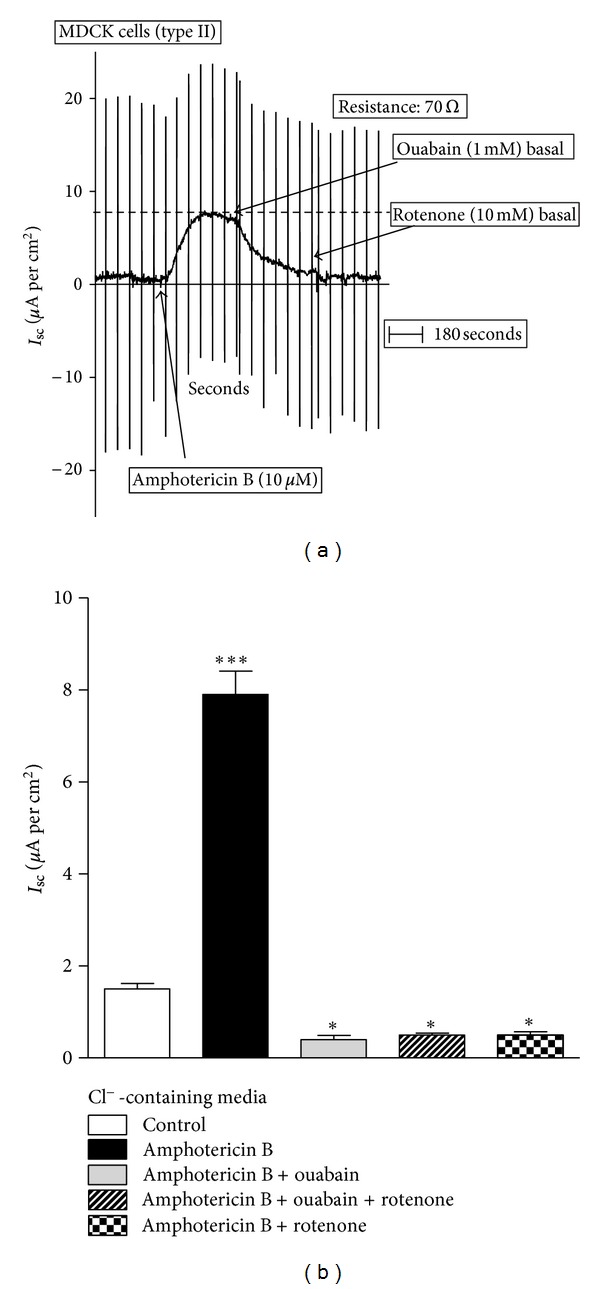
Short-circuit current (*I*_sc_) in permeabilized MDCK cells: Cl^−^-containing media. (a) A typical record is shown to demonstrate the experimental protocol. After equilibration, amphotericin B (10 *μ*M) was added to the apical medium, to permeabilize the apical plasma membrane to small cations. This maneuver allows measuring basolateral pump currents. Then, the effects of ouabain (1 mM) and rotenone (10 *μ*M) were evaluated. (b) Effect of ouabain (1 mM) and rotenone (10 *μ*M) on *I*_sc_ of permeabilized MDCK cells. Results are mean ± SEM of five independent experiments.

**Figure 2:**
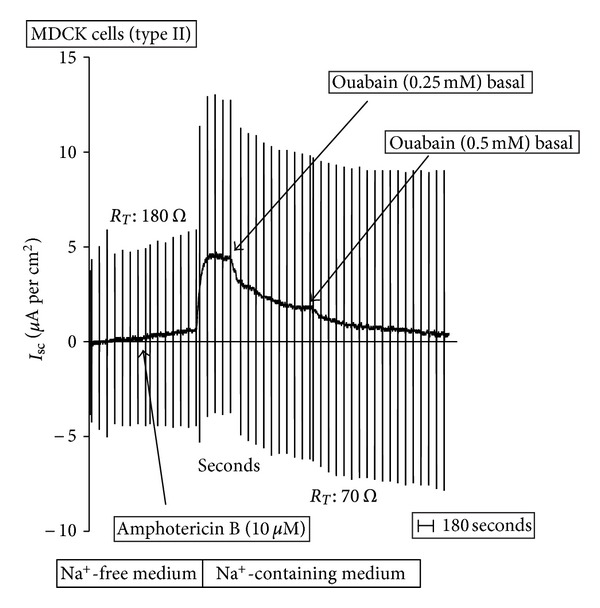
Short-Circuit current (*I*_sc_) in permeabilized MDCK cells: Na^+^-free media. A typical record is shown to demonstrate the experimental protocol. After equilibration in Na^+^-free medium, amphotericin B (10 *μ*M) was added to the apical solution, to permeabilize the apical plasma membrane to small cations. This maneuver allows measuring basolateral pump currents. Then, Na^+^-free media was replaced by Na^+^-containing medium (50 mM) and the effects of ouabain (0.25 and 0.5 mM) was evaluated.

MDCK cell monolayers, cultured on Transwells, were studied under voltage-clamp conditions. As shown in Figure 1(a), under basal conditions, cells showed an *I*_sc_ of 0.95 ± 0.09 *μ*A per cm^2^ with a transepithelial resistance of 70 Ω·cm^−2^. Apical addition of 10 *μ*M amphotericin B, which produces the permeabilization of the apical membrane to small cations, induced a significant increase in *I*_sc_ to 8.1 ± 0.6 *μ*A per cm^2^ without any significant changes in transepithelial conductance. This increase was mostly abolished by the addition of ouabain (1 mM) to the basal side of the epithelium. The subsequent addition of rotenone (10 *μ*M), a metabolic inhibitor, did not produce any further inhibition. Moreover, the addition of rotenone to the preparation before ouabain also abolishes the *I*_sc_ generated by the permeabilization of the apical membrane with amphotericin B (Figure 1(b)). To confirm that amphotericin B exerts its effect increasing intracellular Na^+^ concentration, the effect of the drug was evaluated in MDCK cell monolayers incubated in Na^+^-free media, where Na^+^ was replaced by N-methyl glucamine^+^.  Figure 2 presents a typical experiment where cells were incubated in Na^+^-free medium and *I*_sc_ determined. After a stabilization period, amphotericin B (10 *μ*M) was added to the apical medium. Under this condition, amphotericin B did not produce any significant change in the *I*_sc_. Then, the apical medium was replaced by an Na^+^-containing (50 mM) solution. This change induced a significant increase in *I*_sc_, which was totally inhibited by the addition of 0.5 mM ouabain to the basolateral medium. At lower concentrations, ouabain (0.25 mM) only produced partial inhibition. Notice that in Na^+^-containing media, without amphotericin B, *I*_sc_ is only 1.4 ± 0.11 *μ*A per cm^2^. These results suggest that MDCK cells have only one electrogenic Na^+^-transport mechanism, which is sensitive to ouabain and located at the basolateral membrane. The characteristics of this mechanism are compatible with the Na^+^/K^+^ pump. There is no evidence for electrogenicity of the second sodium pump under the studied conditions. 

**Figure 3:**
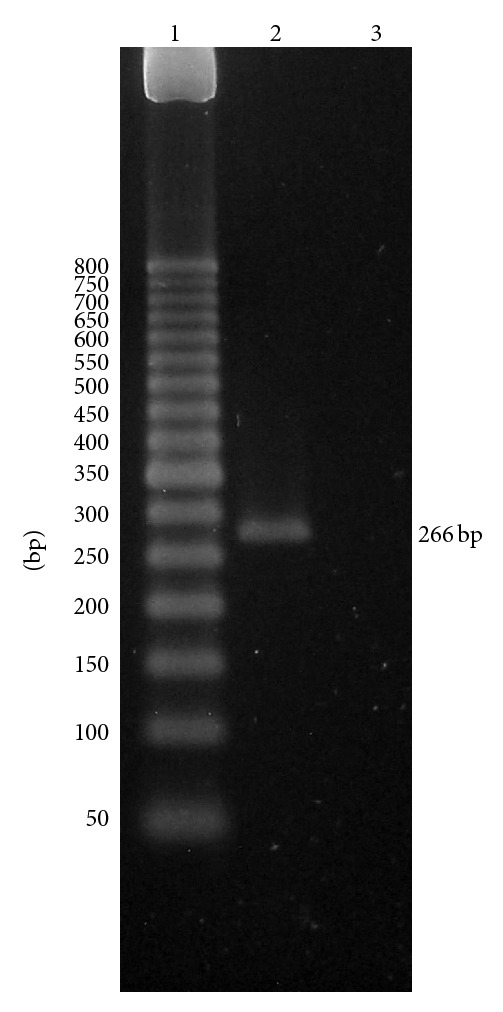
Expression of the Na^+^-ATPase mRNA in MDCK cells by RT-PCR. Expression of the homologous canine ATNA, corresponding to Na^+^-ATPase mRNA, was determined in MDCK cells by specific RT-PCR as described in the methods. PCR products were analyzed by electrophoresis in 2% agarose. The expected *atna* DNA band of 266 bp was amplified from 1 *μ*g of MDCK total RNA (line 2). DEPC-treated water was used as negative control (line 3). The 50 bp DNA ladder from Invitrogen was used as weight marker (lane 1).

One possibility that could explain these results is the absence of the second sodium pump and its associated Na^+^-ATPase in this preparation of MDCK cells. To confirm the existence of the Na^+^-ATPase in MDCK cells, the activity of the K^+^-independent, ouabain-insensitive Na^+^-ATPase activity and its corresponding mRNA *atna* (GI: 170524492) were determined in the preparation. As shown in Table 1, microsomal fraction of MDCK cells has a basal Mg^2+^-dependent ATPase that is stimulated by Na^+^, with additional increase when K^+^ was added to the incubation medium. This K^+^ stimulation was totally inhibited by ouabain which is compatible with the presence of the ubiquitous Na^+^/K^+^-ATPase. However, around a third of the Na^+^-stimulated ATPase was independent of K^+^, insensitive to ouabain, and totally inhibited by 3 mM furosemide. These data indicate that the microsomal fraction of MDCK cells has an ATPase activity compatible with the K^+^-independent and ouabain-insensitive Na^+^-ATPase. The expression of the Na^+^-ATPase *α*-subunit mRNA (atna) was verified in the MDCK cells under the experimental conditions described here, such as what was previously reported [16]. As shown in Figure 3, a specific DNA band of around 266 bp was detected in the MDCK cells (lane 2) without any amplification from the PCR negative control (lane 3). The ouabain-insensitive Na^+^-ATPase from microsomal fraction of MDCK cells has been previously characterized [22]. This is a magnesium-dependent ATPase, specifically stimulated by Na^+^ and inhibited by furosemide and vanadate. In addition, the enzyme is able to generate a phosphorylated intermediate. The specific stimulation by Na^+^, the inhibition by vanadate, and the formation of phosphorylated intermediary define this enzyme as a “P-type” ATPase.

**Figure 4:**
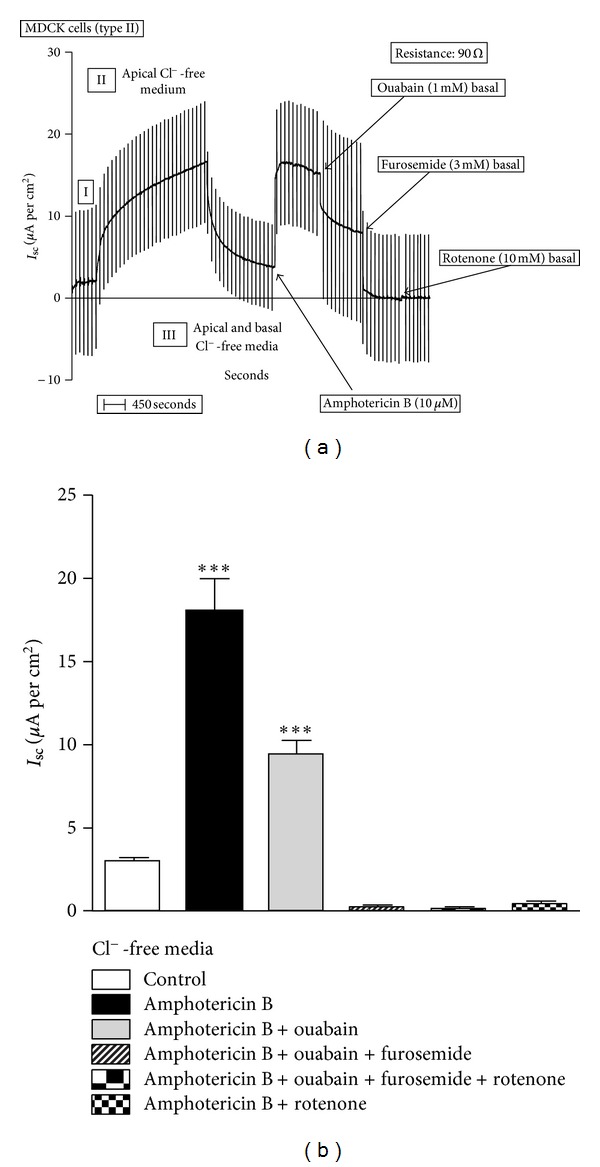
Short-circuit current (*I*_sc_) in permeabilized MDCK cells: Cl^−^-free media. (a) A typical record is shown to demonstrate the experimental protocol. After equilibration (I), Cl^−^ was sequentially removed from the apical (II) and basal sides (III) of the monolayer. Then, amphotericin B (10 *μ*M) was added to the apical medium, to permeabilize the apical plasma membrane. Next, the effects of ouabain (1 mM), furosemide (3 mM), and rotenone (10 *μ*M) were evaluated. (b) Effect of ouabain (1 mM), furosemide (3 mM), and rotenone (10 *μ*M) on *I*_sc_ of permeabilized MDCK cells. Results are mean ± SEM of five independent experiments.

Once the expression of Na^+^-ATPase was confirmed in this cell preparation, it emerges the possibility that the second sodium pump works as an electroneutral mechanism, coupling the active movement of sodium with a passive movement of Cl^−^ through a conductive pathway, resulting in a stoichiometric Na^+^ and Cl^−^ transport across the basolateral plasma membrane of the cells. To evaluate this hypothesis, the experiments were performed in media where Cl^−^ was replaced by gluconate, a less permeable anion. In this case, cells were cultivated in Cl^−^-containing medium, and after the monolayer was 100% confluent, it was studied under voltage-clamp conditions, as described before. Results are shown in Figure 4. Under initial conditions, the *I*_sc_ was of 2.0 *μ*A per cm^2^ and the transepithelial resistance was 90 Ω·cm^−2^. As expected, the Cl^−^ substitution by gluconate in the apical medium induced an increase in the *I*_sc_ to 16.0 *μ*A per cm^2^ due to the diffusional flux of Cl^−^ from the basolateral compartment to the apical one without changes in the transepithelial resistance. The substitution of Cl^−^ in both sides returned the *I*_sc_ to 3.0 *μ*A per cm^2^ without changes in the transepithelial resistance. Under these conditions, the apical membrane was permeabilized to small cations by the addition of 10 *μ*M amphotericin B, inducing an increase of *I*_sc_ to 16.4 *μ*A per cm^2^. The addition of ouabain (1 mM) reduced the *I*_sc_ in 50% to 8.2 *μ*A per cm^2^. Subsequent addition of furosemide (3 mM) abolished the *I*_sc_ in the Cl^−^-free media. After furosemide, the addition of rotenone did not produce any additional effect on the *I*_sc_. Rotenone (10 *μ*M) alone totally inhibited the *I*_sc_ induced by amphotericin B permeabilization (Figure 4(b)). The use of rotenone before the amphotericin B reduced the *I*_sc_ to zero and the subsequent permeabilization with the ionophore did not induce any current (data not shown). These results demonstrate that the second sodium pump is an electroneutral mechanism resulting from the stoichiometry movement of Na^+^ and Cl^−^ across the basolateral plasma membrane of the epithelial cell.

## 4. Discussion

In the proximal tubule and the small intestine, transepithelial sodium transport involves Na^+^ entry across the apical membrane of the epithelial cell and the active exit through the basolateral membrane by two Na^+^ pumps: the Na^+^/K^+^ pump, which exchanges internal Na^+^ by external K^+^, is inhibited by ouabain and is insensitive to furosemide; the second sodium pump, which extrudes Na^+^ accompanied by Cl^−^ and water (for review, see [17]). It has been well established that the Na^+^/K^+^ pump is electrogenic due to the exchange of 3 internal Na^+^ by 2 external K^+^ [23]. The net movement of charges is not compensated by any passive movement of other ions across the plasma membrane. In the case of the second sodium pump, it has been proposed that the pump extrudes sodium by an electrogenic mechanism that induces the passive movement of Cl^−^ to compensate the charge movement. The transport of NaCl would promote the osmotic transfer of water across the membrane and in this way regulate cell volume under isotonic conditions [7, 8, 18].

The electrogenicity of these Na^+^ pumps can be evaluated in epithelial cell monolayers, grown on Transwell and evaluated in the Ussing chambers under voltage-clamp conditions. This preparation represents a model of epithelium where the permeabilization of the apical membrane by ionophores permits the characterization of the electrical parameters of ion transport mechanisms located at the basolateral plasma membrane of the cell. Thus, the electrogenicity of the Na^+^/K^+^ pump has been evaluated in different cultured epithelial cells [24, 25]. An important element in this model is the choice of the ionophore. For instance, amphotericin B is a natural antibiotic product of *Streptomyces nodosus* with fungicidal activity. This polyene molecule is also able to interact with the membranes of eukaryotic cells, forming aqueous pores or nonaqueous (cation-selective) channels. The formation of aqueous or nonaqueous pores depends on (a) the effective concentration of the drug and (b) the type of sterol in the membrane. The presence of ergosterol or cholesterol in the cell membrane determines the concentration at which aqueous or nonaqueous pores are formed. Lower concentrations are required to form nonaqueous pores. Membranes that contain ergosterol require lower amphotericin B concentration to form aqueous pores. The point has been recently reviewed by Cohen [26]. The capacity of polyene antibiotics (amphotericin B and nystatin) to form nonaqueous (cation-selective) channels in eukaryotic membranes has been used, since 1979, to selectively permeabilize the apical or basolateral plasma membrane of epithelial cells from a wide variety of tissues of different species, including cultured epithelial cells [24–32]. In 1986, Harvey and Lahlou [28] evaluated in detail, using intracellular microelectrodes, the effect of amphotericin B on the electrical resistances of the cell membrane and the paracellular pathway and the intracellular ionic activities in trout urinary bladder. Amphotericin B, added to the apical side of the epithelium, increases the transepithelial potential and the short-circuit current. The increases were sensitive to ouabain and dependent on apical Na^+^ concentration. The apical membrane potential depolarized and its resistance fell significantly. At the same time, amphotericin B induced increase in intracellular Na^+^ activity and decrease in K^+^ activity with a small change in Cl^−^ intracellular activity, in agreement with their passive redistribution down their electrochemical gradients. These results demonstrate that the permeabilization of the apical membrane of epithelial cells by amphotericin B induces increase in intracellular Na^+^ and decrease in intracellular K^+^ concentrations. Additionally, they confirmed that the effect of the drug was Na^+^ dependent. Moreover, it was established that the increase in the transepithelial potential was due to the increase in the cell membrane electromotive forces, associated with the activation of the Na^+^/K^+^ pump. The Na^+^ dependence [33] and ouabain sensitivity [24] of the amphotericin B effects on epithelial electrical parameters have also been confirmed in cultured cells.

In the present study, using this model, the electrogenicity of the Na^+^/K^+^ pump was confirmed but the second sodium pump seems not to be electrogenic when Cl^−^ is present in the medium. Under these conditions, all the current generated by the pumps across the basolateral plasma membrane of the MDCK cells is due to the Na^+^/K^+^ pump, since it is totally inhibited by 1 mM ouabain (Figure 1). The electrogenicity of the second sodium pump can be demonstrated only in the absence of the chloride. Under these conditions, the existence of a furosemide-sensitive *I*_sc_ current, in addition to an ouabain-sensitive one, was identified (Figure 4). The inhibition of the amphotericin B-induced *I*_sc_ by rotenone indicates that both mechanisms depend on cellular metabolism. Moreover, cyanide, a metabolic inhibitor that blocks the cytochrome oxidase, resembles the effect of rotenone on the amphotericin B-induced *I*_sc_ (data not shown). However, it is possible that rotenone could directly inhibit the Na^+^- and Na^+^/K^+^-ATPases. To discard this possibility, the effect of the drug on the ATPase activities present in a microsomal fraction of MDCK cells was evaluated. Membranes were incubated with rotenone (10 *μ*M) for 20 minutes at 4°C and the ATPases activities were determined as indicated in the methods. Rotenone has no effects on the ATPases present in the microsomal fraction. 

The passive Cl^−^ movement associated with the function of the second sodium pump seems to be regulated by the action of the pump itself. Thus, an electroneutral transport mode of the second sodium pump is observed in the presence of Cl^−^ in the medium. In contrast, in the absence of this anion, the second sodium pump seems to be electrogenic, since a furosemide-sensitive *I*_sc_ becomes evident. These observations raise the question about how the Cl^−^ movement is linked to the second sodium pump function. Chloride ions could move through an independent conductive pathway or form an integral element of the pump. In this sense, there are several lines of evidences indicating that the second sodium pump function does not require Cl^−^. The hydrolytic activity of the furosemide-sensitive Na^+^-ATPase is independent of the anion accompanying sodium [11, 16]. In addition, the ATP-dependent Na^+^ transport through the second sodium pump, in inside-out membrane vesicles [10] and isolated cells [8], is not affected by the total replacement of Cl^−^ in the incubation medium, indicating that the function of the Na^+^-ATPase do not depend on the presence of Cl^−^ in the medium. Moreover, the modeled 3D structure of the Na^+^-ATPase, based on ATNA homology with other P-type ATPases, lacks transmembrane domains that resemble a chloride channel [17]. These evidences support the hypothesis that no Cl^−^ conductive pathway forms a part of the second sodium pump. However, Cl^−^ transport associated with the second pump has been demonstrated in renal tissue and intestinal isolated cells [7, 8]. Thus, renal and small intestinal epithelial cells are able to actively extrude Na^+^, Cl^−^, and water, even in the presence of ouabain. In contrast, the Na^+^ extrusion, mediated by the Na^+^/K^+^ pump, does not induce any Cl^−^ nor water extrusion. These results demonstrate that a Cl^−^ movement is associated with the active Na^+^ transport mediated by the second sodium pump but the anion is not necessary to its function. The conductive movement of Cl^−^ through the basolateral membrane of the epithelial cell seems regulated by the second sodium pump. An independent conductive pathway for Cl^−^ should also affect the electrogenicity of the Na^+^/K^+^ pump, but it is not the case in our experiments. The extent of the ouabain-sensitive *I*_sc_, an expression of the Na^+^/K^+^ pump, is quite similar in the presence and absence of Cl^−^ in the medium (Figures 1, 3, and 4), indicating that the electrogenicity of this pump is not affected by the presence of chloride. Finally, *I*_sc_ was also evaluated in cell monolayers preincubated with furosemide (3 mM) and incubated in the absence of Cl^−^ in the medium. Results were very similar to that observed in Cl^−^-containing media. The *I*_sc_ induced by amphotericin B permeabilization was totally inhibited by ouabain and its extend (6.5 ± 0.25 *μ*A per cm^2^) was comparable to that obtained in Cl^−^-containing media, supporting the view that the *I*_sc_ mediated by the Na^+^/K^+^ pump is not modified by the absence of Cl^−^ in the medium. Our results demonstrate that the second sodium pump is an electroneutral mechanism resulting from the stoichiometry Na^+^ and Cl^−^ movement across the basolateral plasma membrane of the epithelial cell.

## Figures and Tables

**Table 1:** ATPase activities of microsomal fraction from MDCK cells.

Conditions	Mg^2+^	Mg^2+^ + Na^+^	Mg^2+^ + Na^+^ + K^+^
Control	49.25 ± 1.65	93.29 ± 2.42	183.88 ± 10.31
Ouabain	49.52 ± 2.64	90.73 ± 3.33	95.76 ± 4.20
Ouabain + furosemide	52.94 ± 3.06	53.26 ± 3.30	55.54 ± 4.12

Results are expressed in nmoles Pi Lib./min∗mg of protein and represent mean ± SEM of seven independent preparations.
